# Signet-ring cell colon carcinoma in a Northern Ghanaian child: a case report

**DOI:** 10.1097/RC9.0000000000000359

**Published:** 2026-04-02

**Authors:** German Azahares Leal, Ramon Andres Ramírez Calas, Tania González Millán, Evans Degbor

**Affiliations:** a Department of Surgery, Tamale Teaching Hospital, Tamale, Ghana; b Department of Surgery, School of Medicine and Health Science, University for Development Studies, Tamale, Ghana; c Pathology Department, Tamale Teaching Hospital, Tamale, Ghana; d Department of Anatomy, School of Medicine and Health Science, University for Development Studies, Tamale, Ghana

**Keywords:** case report, early-onset CRC, mismatch repair proficiency, pediatric colorectal cancer, signet ring cell carcinoma

## Abstract

**Introduction and importance:**

Colorectal cancer (CRC) in children represents less than 1% of pediatric malignancies worldwide. Signet-ring cell carcinoma (SC), a subtype associated with mucin-producing phenotypes and distinct molecular pathways, is infrequently encountered in this age group, particularly in low-resource settings.

**Presentation of case:**

A 10-year-old boy presented with 1 month of right lower quadrant pain, progressive abdominal distension, anorexia, constipation, and weight loss. Radiologic studies (plain abdominal X-ray, barium enema, and CT scan) showed right-sided colonic obstruction with secondary compression of the duodenum and gallbladder, initially raising suspicion of congenital duodenal bands. Laparotomy identified obstructing mass at the hepatic flexure. Right hemicolectomy was performed. Histology confirmed poorly differentiated SC (pT4a N2b M0) with extensive lymphovascular invasion. Immunohistochemistry indicated mismatch repair proficiency.

**Clinical discussion:**

Pediatric SC CRC is only sparsely reported, and available data suggest distinct biological characteristics. The tumor’s diffuse infiltrative pattern is typically driven by intracellular mucin accumulation that displaces the nucleus, loss or reduction of E-cadherin–mediated cell adhesion, and activation of epithelial–mesenchymal transition pathways. Mismatch repair stability, as observed in this case, points toward non–MMR-driven oncogenesis, indicating a carcinogenic pathway independent of Lynch syndrome or sporadic MSI-high mechanisms. MMR-stable early-onset tumors may develop via chromosomal instability, epigenetic promoter methylation, or Wnt/β-catenin signaling activation.

**Conclusion:**

This case illustrates the complex molecular features characterizing signet-ring cell colorectal carcinoma in young patients reinforcing the need for heightened diagnostic vigilance and further region-specific studies to better delineate the underlying biology of early-onset CRC in resource-limited settings.

## Introduction

Colorectal cancer (CRC) is exceptionally rare in the pediatric population, accounting for less than 1% of all childhood malignancies worldwide^[^1,2^]^. Diagnosis is often delayed due to nonspecific symptoms, low clinical suspicion, and the prevailing view that CRC is a disease of adulthood. When it does occur in children, it usually presents at an advanced stage and with aggressive histological subtypes, resulting in poor prognosis^[^3^]^.

HIGHLIGHTS

- Signet ring cell carcinoma of the colon is exceptionally rare in pediatric patients.
- We report a 10-year-old boy with advanced signet ring cell colorectal carcinoma in Northern Ghana.
- Imaging showed colonic obstruction with secondary duodenal and gallbladder compression.
- This case illustrates the complex molecular features characterizing signet-ring cell colorectal carcinoma in young patients
- Underscores the need for greater clinical awareness, earlier detection, and dedicated researches on early-onset CRC in West Africa.

While pediatric CRC remains uncommon, emerging molecular studies suggest that colorectal and other malignancies in African populations may harbor distinct genetic alterations compared to Western or Asian cohorts^[^4,5^]^. Reports from Ghana and other sub-Saharan countries describe a higher prevalence of mismatch repair-proficient tumors, fewer APC or KRAS mutations, and possible novel germline or somatic variants^[^6^]^. Similar molecular patterns have been observed in breast, prostate, and liver cancers in the region^[^7^]^, suggesting unique cancer pathways influenced by local genetic backgrounds and environmental exposures. These differences highlight the urgency of developing cancer guidelines and screening strategies adapted to regional genomic profiles and resource limitations, rather than relying solely on data from high-income countries.

The present case of a 10-year-old boy diagnosed with advanced signet-ring cell colorectal carcinoma underscores the need for greater clinical awareness, earlier detection, and dedicated researches on early-onset CRC in West Africa.

This case report has been reported in line with the SCARE 2025 checklist^[^8^]^.

### Case summary

A 10-year-old primary school male patient, previously healthy, presented with a 1-month history of intermittent right lower quadrant and periumbilical abdominal pain, progressive abdominal distension, anorexia, constipation, and significant weight loss. The symptoms began after he joined his family in fasting during Ramadan, which initially masked the severity of his reduced oral intake. The pain was described as colicky and was accompanied by occasional non-bilious, projectile vomiting. There was no history of gastrointestinal bleeding, allergies, or drug consumption for any chronic illness. Family history of colorectal cancer or inflammatory bowel disease was not neither taken.

Physical examination revealed a chronically ill, cachectic child with periorbital edema and a grossly distended, tense, but non-tender abdomen. Bowel sounds were high-pitched and hyperactive. Digital rectal examination showed normal anal tone, no palpable masses, and scanty dark stool.

Laboratory studies were largely unremarkable, including hemoglobin of 13 g/dl and normal renal/liver function tests.

**Figure 1.**
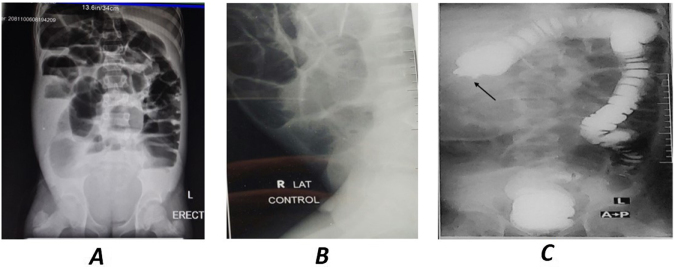
Dilated and edematous Small bowel loops + airfluid levels. (A) Plain abdominal X-ray (control). PA erect view. (B) PA lateral view. (C) Barium Enema Abd X Ray (AP view). Abrupt termination and narrowing of hepatic flexure, giving the “apple core” deformity.

Imaging studies included a plain abdominal X-ray, Barium enema, and abdominal CT scan. These showed marked distension of the distal Ileum/ right colon, with an abrupt transition point at the hepatic flexure, suggesting an obstructive lesion (Fig. 1).

**Figure 2.**
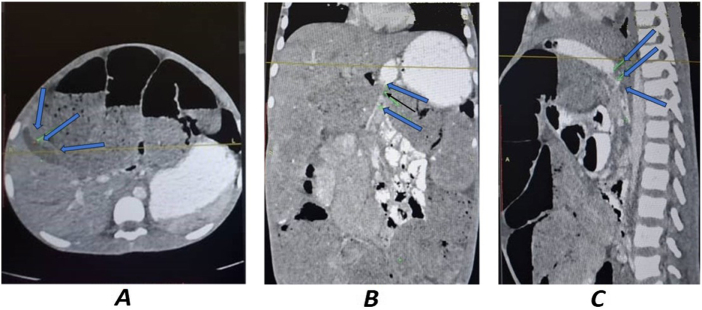
Gallblader and duodenal compression. (A) CT-scan axial view (gallblader compression, large bowel loops edematous and dilated). (B) CT-scan coronal view (duodenal compression). (C) CT-scan sagittal view (duodenal compression, large bowel loops edematous and dilated).

Importantly, the CT scan also demonstrated secondary compression of adjacent structures: the duodenum and gallbladder appeared displaced and compressed by the massively distended colon and tumor bulk (Fig. 2).

**Figure 3.**
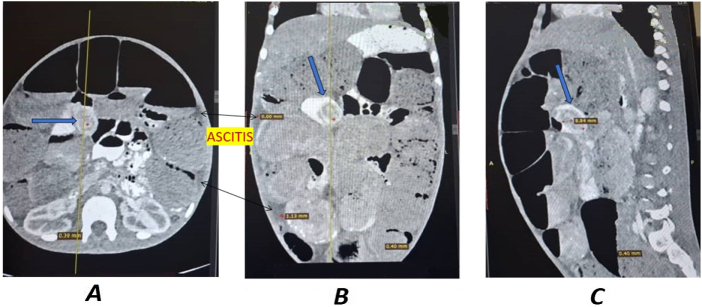
Hepatic flexure colonic mass, large bowel loops edematous and dilated. (A) CT-scan axial view. (B) CT-scan coronal view. (C) CT-scan sagittal view.

These findings initially raised suspicion of an extrinsic cause of obstruction, such as congenital duodenal bands, particularly given the patient’s age and the absence of a clearly visualized mass at first glance. However, further review of the images supported the presence of a colonic mass as the primary etiology (Fig. 3).

**Figure 4.**
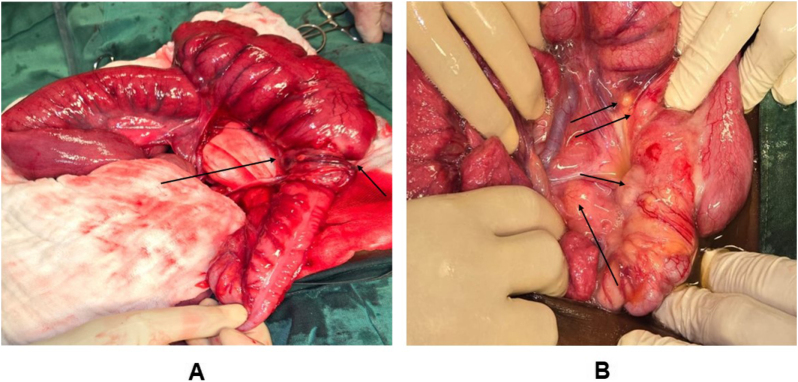
(A) Obstructive tumor involving the colonic hepatic flexure. (B) Multiple lymph nodes.

Once all diagnostic evidence had been gathered, elective surgery was scheduled 5 days after admission. Prophylactic antibiotics for colonic surgery were administered, while antegrade mechanical bowel preparation was omitted due to the risk of obstruction. The patient was in good general condition and was managed by a multidisciplinary team comprising a pediatric surgeon and a colorectal surgeon, both senior consultants with more than 30 years of experience, assisted by second-year general surgery residents. Under general endotracheal anesthesia, the abdominal cavity was accessed through a midline supra- and infraumbilical incision. During laparotomy, the liver and the remainder of the abdominal cavity were thoroughly explored, with no remarkable findings. A large obstructing mass was identified at the hepatic flexure and proximal transverse colon, exhibiting dense adhesions to adjacent structures, including the duodenum and gallbladder, which explained the radiological impression of external compression. A right hemicolectomy was performed, encompassing resection of the terminal ileum, cecum, ascending colon, and the proximal two-thirds of the transverse colon, followed by a primary ileocolic anastomosis (Fig. 4).

**Figure 5.**
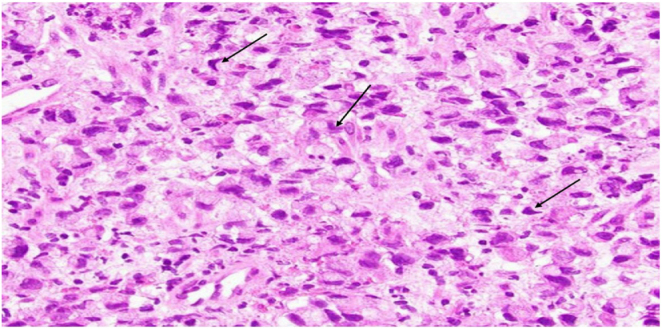
Signet-ring cell carcinoma.

*Postoperative Histopathological examination* revealed a poorly differentiated signet-ring cell carcinoma, diffuse type (Fig. 5), invading the full thickness of the colonic wall and visceral peritoneum, with extensive lymphovascular invasion. Of 21 lymph nodes examined, 15 were positive for metastases, corresponding to a pathological stage of pT4a N2b M0. According to the AJCC 8th edition staging system, the final pathological stage was *Stage IIIC (pT4aN2bM0).*

*Immunohistochemical analysis* was performed, including mismatch repair (MMR) proteins (MLH1, MSH2, MSH6, PMS2) and BRAF V600E mutation testing. All four MMR proteins showed preserved nuclear expression, indicating MMR proficiency (microsatellite stable status). BRAF mutation testing was negative.

The postoperative course was uneventful, and the patient was discharged on the fifth postoperative day. He attended two follow-up consultations at our tertiary teaching hospital during the first month. Subsequently, he was referred to the pediatric oncology unit for evaluation and planning of adjuvant therapy, where he is currently undergoing treatment. Genetic counseling was also offered to the family, with recommendations for first-degree relatives to undergo appropriate screening aimed at early detection and intervention should a hereditary predisposition be present. However, we acknowledge the significant financial constraints faced by many families in our setting, which often limit access to screen testing. To address this gap, we are actively seeking to include such high-risk relatives in ongoing and future locally driven research and outreach projects targeting vulnerable populations.

## Discussion

Signet-ring cell (SC) adenocarcinoma is a rare histological subtype of colorectal cancer (CRC), accounting for approximately 1% of cases and characterized by abundant intracytoplasmic mucin and diffuse stromal infiltration^[^9,10^]^. Excess mucin facilitates tumor spread along tissue planes, while reduced E-cadherin expression impairs epithelial adhesion, promoting the infiltrative pattern and early dissemination typical of this variant^[^11^]^. These biological features are consistent with the extensive lymphovascular invasion and advanced stage observed in the present case.

The tumor demonstrated mismatch repair (MMR) proficiency, indicating a carcinogenic pathway independent of Lynch syndrome or sporadic MSI-high mechanisms. MMR-stable early-onset tumors may develop via chromosomal instability, epigenetic promoter methylation, or Wnt/β-catenin signaling activation^[^12–15^]^. These mechanisms, increasingly reported in young CRC patients without hereditary syndromes, highlight the heterogeneity of non-MMR-driven carcinogenesis and support the need for molecular characterization in pediatric cases.

Initial radiological evaluation suggested duodenal and perihepatic compression compatible with congenital anomalies such as Ladd’s bands. This emphasizes the diagnostic complexity of pediatric presentations with nonspecific obstructive symptoms. Subsequent imaging localized the pathology to a right-sided colonic mass, underscoring the importance of considering malignancy even when congenital explanations appear plausible.

Reports from multiple centers in West Africa indicate increasing identification of early-onset CRC, frequently presenting at advanced stages and commonly MMR-proficient^[^16,17^]^. Although comprehensive population-level data remain limited, institutional observations suggest a higher prevalence than previously recognized^[^16^]^. Environmental and infectious cofactors are under investigation. Chronic intestinal schistosomiasis has been proposed as one such contributor, as persistent mucosal inflammation, oxidative DNA damage, and fibrosis may create a microenvironment conducive to malignant transformation^[^18–20^]^.

A prior case from the same institution described a similar pediatric presentation without identifiable hereditary basis^[^21^]^, reinforcing the need for improved recognition and systematic study of pediatric CRC in the region^[^16^]^.

This case underscores the importance of maintaining a broad differential diagnosis in children with persistent or atypical abdominal symptoms. Strengthening national cancer registries, expanding access to diagnostic endoscopy, and developing local capacity for molecular and genomic analysis are essential for characterizing the biological and epidemiological features of early-onset CRC in sub-Saharan Africa. Systematic documentation of these cases will inform diagnostic strategies and support multicenter research initiatives^[^22^]^.

## Conclusion

Pediatric signet-ring cell colorectal carcinoma is exceedingly rare, yet this case underscores the need to consider malignant etiologies in children presenting with bowel obstruction and to employ early, targeted imaging. Although reports from sub-Saharan Africa describe colorectal cancer in younger patients, the available evidence does not yet support a confirmed epidemiologic trend, highlighting the need to strengthen regional cancer registries and systematic reporting. Overall, this case emphasizes the importance of timely diagnostic pathways and access to molecular testing to better characterize these uncommon tumors and optimize management, particularly in resource-limited settings.

## Data Availability

The data that support the findings of this study are available from the corresponding author upon reasonable request.

## Patient perspective

Throughout the course of his treatment, the parents expressed satisfaction with the care their child received.
